# Correction to “Ambient Measurements of Hazardous Air Pollutants in the United States Routinely Exceed Predictions from Screening-Level Exposure Models”

**DOI:** 10.1021/acs.estlett.5c00378

**Published:** 2025-05-08

**Authors:** Lauren E. Padilla, Daniel R. Peters, Elizabeth J. Mohr, Ramón A. Alvarez

The corrected Figure 1 includes the complete set of panels for the 10 chemicals described in the caption and main text. The originally published figure omitted panels for ethylene oxide and chloroprene. This correction does not affect the results or conclusions of the paper. No changes were made to the caption, the information displayed for other chemicals, or any of the underlying data for Figure 1, all of which can be found in Supporting Information Table S2 of the published paper.

**Figure 1:**
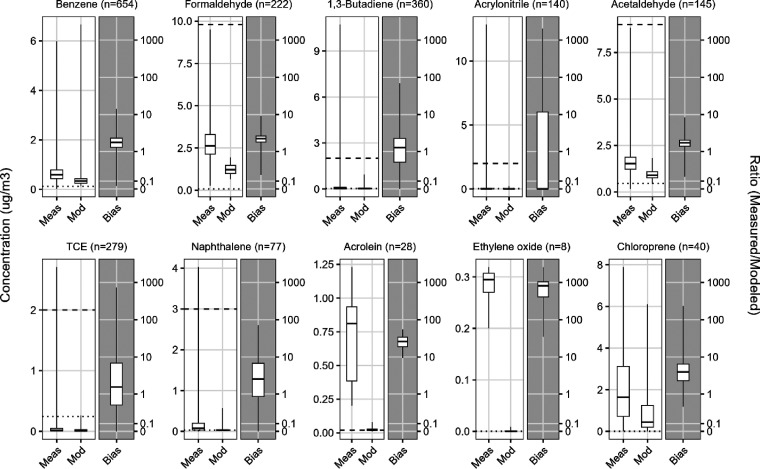
Comparison of modeled (Mod) and measured (Meas) concentrations for 10 chemicals of top health concern. For each chemical, the left chart shows nationwide annual average concentration distributions, including all years of data (2017–2019), if available, with sample size (i.e., number of monitors times years) *n*, relative to health thresholds: the non-cancer inhalation reference concentration (dashed line) and the concentration estimated to pose 1-in-1 million lifetime excess cancer risk (dotted line). The non-cancer reference is not shown for benzene, ethylene oxide, or chloroprene because it exceeds the vertical axis upper bound. The cancer risk threshold is not shown for acrolein, which lacks a cancer assessment. The right chart shows the distribution of the corresponding bias, defined as the ratio of measured to modeled concentration. The bias is plotted on a pseudo-logarithmic scale that transitions to linear at 0.1 to allow inclusion of ratio values equal to zero. All box plot whiskers extend to minimum and maximum points, and the box depicts the 25th, 50th, and 75th percentiles. TCE = trichloroethylene. Corresponding scatter plots of measured and modeled concentrations are provided in Supporting Information Figure S2.

